# Documenting the immune response in patients with COVID-19-induced acute respiratory distress syndrome

**DOI:** 10.3389/fcell.2023.1207960

**Published:** 2023-06-09

**Authors:** Junyu Lu, Xiaona Zeng, Weisheng Lu, Jihua Feng, Yegui Yang, Yongxian Wei, Yin Chen, Jianfeng Zhang, Liao Pinhu

**Affiliations:** ^1^ Intensive Care Unit, The Second Affiliated Hospital of Guangxi Medical University, Nanning, China; ^2^ Department of Emergency Medicine, The Second Affiliated Hospital of Guangxi Medical University, Nanning, China

**Keywords:** COVID-19, acute respiratory distress syndrome, single-cell RNA sequencing, immune cells, dysregulated immune response

## Abstract

**Introduction:** Coronavirus disease 2019 (COVID-19) can lead to acute respiratory distress syndrome (ARDS) and life-threatening multi-organ failure with increased levels of inflammatory mediators and viral load; however, little is known about its pathophysiology.

**Methods:** To better understand the cellular status of COVID-19-induced ARDS, we performed single-cell RNA sequencing on peripheral blood samples from patients with COVID-19-induced ARDS. Single-cell RNA sequencing combined with bioinformatics analysis was used to study dynamic changes in cell composition and transcriptional profiles.

**Results:** The single-cell RNA sequencing data revealed significant phenotypic differences between patients with COVID-19-induced ARDS and controls, mainly in monocytes, and CD8^+^ T and B cells. B-cell and monocyte abundances were significant in COVID-19-induced ARDS patients compared to controls, while CD8^+^ T cells were depleted. These data suggest that there is an imbalance between lymphocytes and monocytes in the blood of COVID-19-induced ARDS patients. In addition, cytokine interactions between T cells, monocytes and B cells are enhanced as evidenced by the intercellular communication analysis. In particular, T cell subsets target receptors on other cells via CCL5 and may play an important role in patients with COVID-19-induced ARDS.

**Conclusion:** Our analysis suggested that a dysregulated adaptive immune response exists in patients with COVID-19-induced ARDS. Overall, we provided a cellular picture of the peripheral immune response in patients with COVID-19-induced ARDS.

## 1 Introduction

Most patients with severe acute respiratory syndrome coronavirus 2 (SARS-CoV-2) present with benign flu-like symptoms which can be effectively controlled (Chen et al., 2020). Some patients develop severe systemic inflammation with acute respiratory distress syndrome (ARDS), which often leads to multi-organ failure and death (Wu and McGoogan, 2020). Innate immune cells are the first line of defense against invading viruses (Kawai and Akira, 2006), and the vast majority of patients with COVID-19-induced ARDS exhibit high levels of cytokines and chemokines (Hadjadj et al., 2020). COVID-19-induced ARDS may be caused by an increased inflammatory response, an innate immune cell-mediated cytokine storm, sustained viral load, or defective antiviral defense pathways (Hadjadj et al., 2020; Pedersen and Ho, 2020; Wang et al., 2020; Yan et al., 2020; Galani et al., 2021). A previous study found reduced lymphocyte counts in patients with COVID-19 (Zhang et al., 2020a; Wen et al., 2020) and an abundance of pro-inflammatory monocytes in the peripheral blood mononuclear cells (PBMCs) of patients with COVID-19 (Hasan et al., 2021); however, the contribution of these conditions to ARDS remains unknown.

COVID-19-induced ARDS requires prolonged mechanical ventilation or extracorporeal membrane pulmonary oxygenation and is associated with high mortality (Barbaro et al., 2020). Clarification of pathophysiology of COVID-19-induced ARDS is essential for improving disease treatment. To understand the immune status of the blood of patients with COVID-19-induced ARDS, we collected PBMCs from healthy controls and patients with COVID-19-induced ARDS, and performed high-resolution single-cell RNA sequencing (scRNA-seq) of all immune cell subpopulations.

This study aimed to determine the usefulness of scRNA-seq in patients with COVID-19-induced ARDS and to provide a cellular picture of the peripheral immune response.

## 2 Materials and methods

### 2.1 Patients

Our study was conducted in accordance with the Helsinki Declaration and was approved by the Ethics Committee. We included five healthy control donors and eight patients with COVID-19-induced ARDS who did not meet any of the following exclusion criteria (Supplementary Table S1). Peripheral blood sampling was performed on all participants. The following exclusion criteria was followed: a significant history of hematologic malignancy or bone marrow disease, HIV infection, on immunosuppressive medication, or were a solid organ transplant recipient. Informed consent was obtained from all patients participating in the study.

### 2.2 PBMC collection and processing

Human peripheral blood (20 mL) was collected in ethylenediaminetetraacetic acid anticoagulation tubes and centrifuged at 2000 rpm for 10 min at room temperature to separate the plasma. To each tube, we: 1) added 5 mL of lymphocyte separation solution (Wu and McGoogan, 2020); aspirated the upper layer of plasma after centrifugation (Kawai and Akira, 2006); then added an equal volume of sterile phosphate-buffered saline (PBS) solution to dilute the remaining blood cells (Hadjadj et al., 2020); the diluted blood cells were then added to the upper layer of the lymphocyte separation solution in a 1:1 ratio, and centrifuged at 2,500 rpm for 20 min. The middle white film layer was aspirated into a new 10 mL centrifuge tube, PBS solution was added and diluted to 10 mL, mixed, and centrifuged at 2000 rpm at room temperature for 5 min. The lymphocyte separation solution was then removed. PBS (1 mL) was added to dilute the resuspended cells; the volume was fixed to 10 mL, centrifuged at 1,200 rpm at room temperature for 5 min, and the supernatant discarded. The separated PBMC precipitate was resuspended in a cell lyophilization solution.

### 2.3 Single-cell RNA-seq library preparation and sequencing

scRNA sequencing libraries were constructed using DNelab C4 and following the manufacturer’s instructions (Liu et al., 2019). Libraries were quantified using a Qubit ssDNA Analysis Kit (Thermo Fisher Scientific) and sequenced using a DIPSEQ T1 sequencer from the China National Gene Bank. Briefly, single-cell suspensions were used for droplet generation, emulsion breakage, bead collection, reverse transcription, and cDNA amplification was used to generate barcoded libraries. Indexed single-cell RNA-seq libraries were constructed according to manufacturer’s protocol. Sequencing libraries were quantified using the QubitTM ssDNA Assay Kit (Thermo Fisher Scientific, #Q10212). DNA nanoballs were loaded into the patterned nanoarrays and sequenced on an ultra-high-throughput DIPSEQ T1 sequencer using the following read lengths: 30 bp for read 1 (inclusive of 10 bp cell barcode 1), 10 bp for cell barcode 2, 10 bp for the unique molecular identifier; 100 bp of transcript sequence for read 2, and 10 bp for the sample index.

### 2.4 Processing of raw scRNA-seq data

We evaluated the violin plot distribution of the number of unique molecular identifiers and the total number of genes detected per cell (nFeatures) in all samples. Cells with fewer than 200 genes were filtered, and cells with more than 10% mitochondrial transcript content were removed. These quality control metrics aids in filtering cells with poor viability and quality.

### 2.5 Integration of individual cell matrices into expression matrices for all samples

The “IntegrateData” function of the Serurat package was used to integrate the dataset (Butler et al., 2018) and perform cell clustering analysis based on the default parameters (http://satijalab.org/seurat/). In the subsequent analysis, the Uniform Manifold Approximation and Projection (UMAP) algorithm was used to downscale the data and visualize the cell clusters (Becht et al., 2018).

### 2.6 Cell type annotation

Cells were manually annotated according to the expression levels of their respective cell type markers. In addition, to decipher the specific alterations occurring in each cell subpopulation, we clustered each cell subpopulation separately and visualized them using UMAP.

### 2.7 Pathway enrichment analysis

Pathway enrichment analysis was performed on cell subtypes to identify the involved enrichment pathways. The Kyoto Encyclopedia of Genes and Genomes (Kanehisa and Goto, 2000; Kanehisa, 2019; Kanehisa et al., 2023) enrichment analyses was performed using the clusterProfiler package (Yu et al., 2012) based on the expression of marker genes, and results with a *p* < 0.05 were considered significant.

### 2.8 Single-cell trajectory analysis

For cell subpopulations, we computed the pseudotemporal inference using the Monocle3 algorithm (https://cole-trapnell-lab.github.io/monocle3) and visualized it using UMAP (Cao et al., 2019). First, genes that expressed less than 10 cells were removed. Second, the data were normalized and centralized. The top 5,000 genes were selected for subsequent analysis, and the important features of the matrix were extracted using the algorithm of partial singular value decomposition to establish the intermediate structure of the high-dimensional space.

### 2.9 Analysis of intercellular communication

We used iTALK (version 0.1.0) to calculate the expression of receptor ligands in each cell subpopulation (Wang et al., 2019), with the cell populations as the object of interaction. We used this as an indicator of the cellular communication between subpopulations.

### 2.10 Statistics and analysis

Statistical analyses were performed using R software (version 4.0.5) for all studies. Differentially expressed genes between the healthy control and disease groups were identified using the “FindAllMarkers” function in Seruat, and differences associated with *p* < 0.05 were considered significant. Comparisons between two groups were performed using the Student’s t-test, and correlation coefficients were calculated using the Spearman analysis.

## 3 Results

### 3.1 Imbalance between peripheral lymphocytes and monocytes in patients with COVID-19-induced ARDS

**FIGURE 1:**
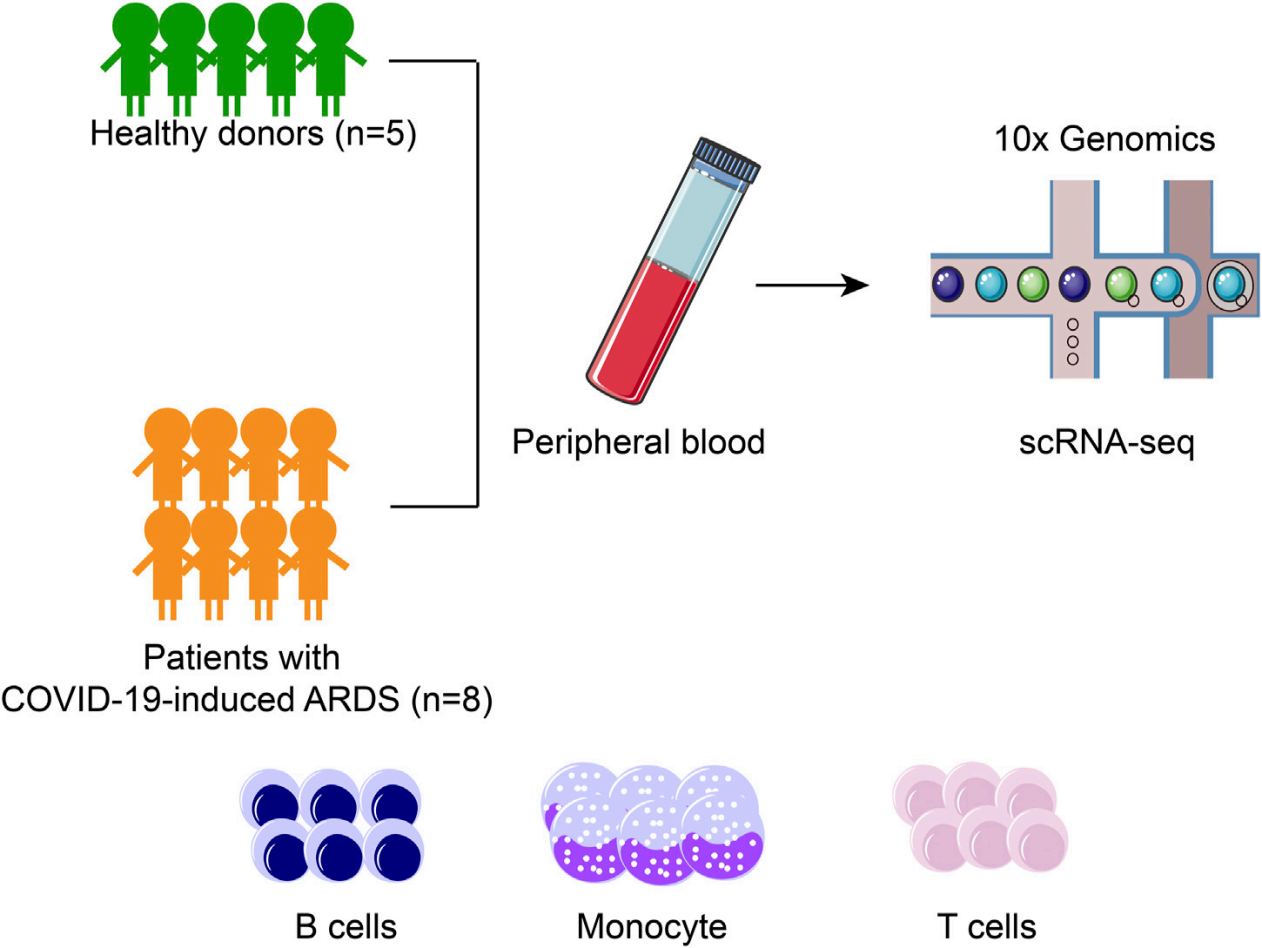
Schematic diagram showing the overall study design.

**FIGURE 2:**
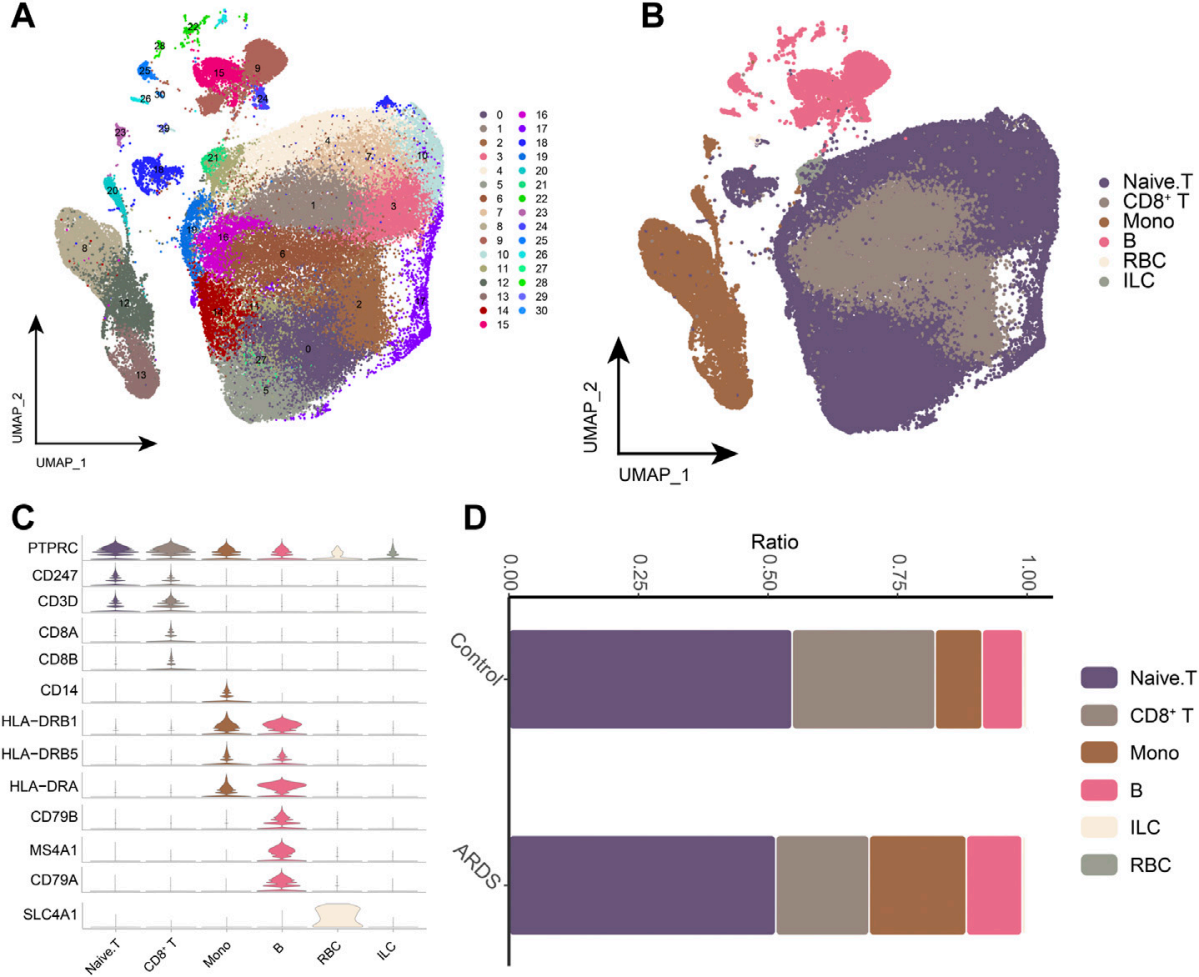
Single-cell transcriptional analysis of peripheral immune cells **(A)**. Single-cell mapping Cluster, a total of 31 cell clusters of 5,318 cells were captured. **(B)**. Single-cell mapping CellType. **(C)**. CellMarker for guiding cell annotation. **(D)**. Differences in cell abundance in PBMC from healthy controls and COVID-19-induced ARDS patients. RBC, red blood cell; ILC, innate lymphoid cell.

We sequenced 85,084 cells with an average of 5,318 cells per sample (Figure 1, Supplementary Table S2). We created a cell expression matrix and performed dimensionality reduction using UMAP to identify 31 clusters (Figure 2A). The highest differentially expressed genes were calculated to annotate the clusters with the respective cell identities: CD8^+^ T cells (CD8A and CD8B), monocytes (Mono) (CD14 and CSF1R) and B cells (CD79A, CD79B, CD19, and MS4A1) (Figures 2B,C; Supplementary Table S3). The UMAP Atlas revealed significant phenotypic differences between patients with COVID-19-induced ARDS and the controls, mainly in monocytes and CD8^+^ T and B cells (Figure 2D). B-cell and monocyte abundances were significant in the patients with COVID-19-induced ARDS compared to the controls, while CD8^+^ T cells were depleted; these data suggest that there is an imbalance between lymphocytes and monocytes in the blood of patients with COVID-19-induced ARDS. We observed an increased abundance of platelets in patients with COVID-19-induced ARDS, which may play a role in clot formation and severe SARS-CoV-2 infection.

### 3.2 Characterization of monocyte subpopulations in patients with COVID-19-induced ARDS

**FIGURE 3:**
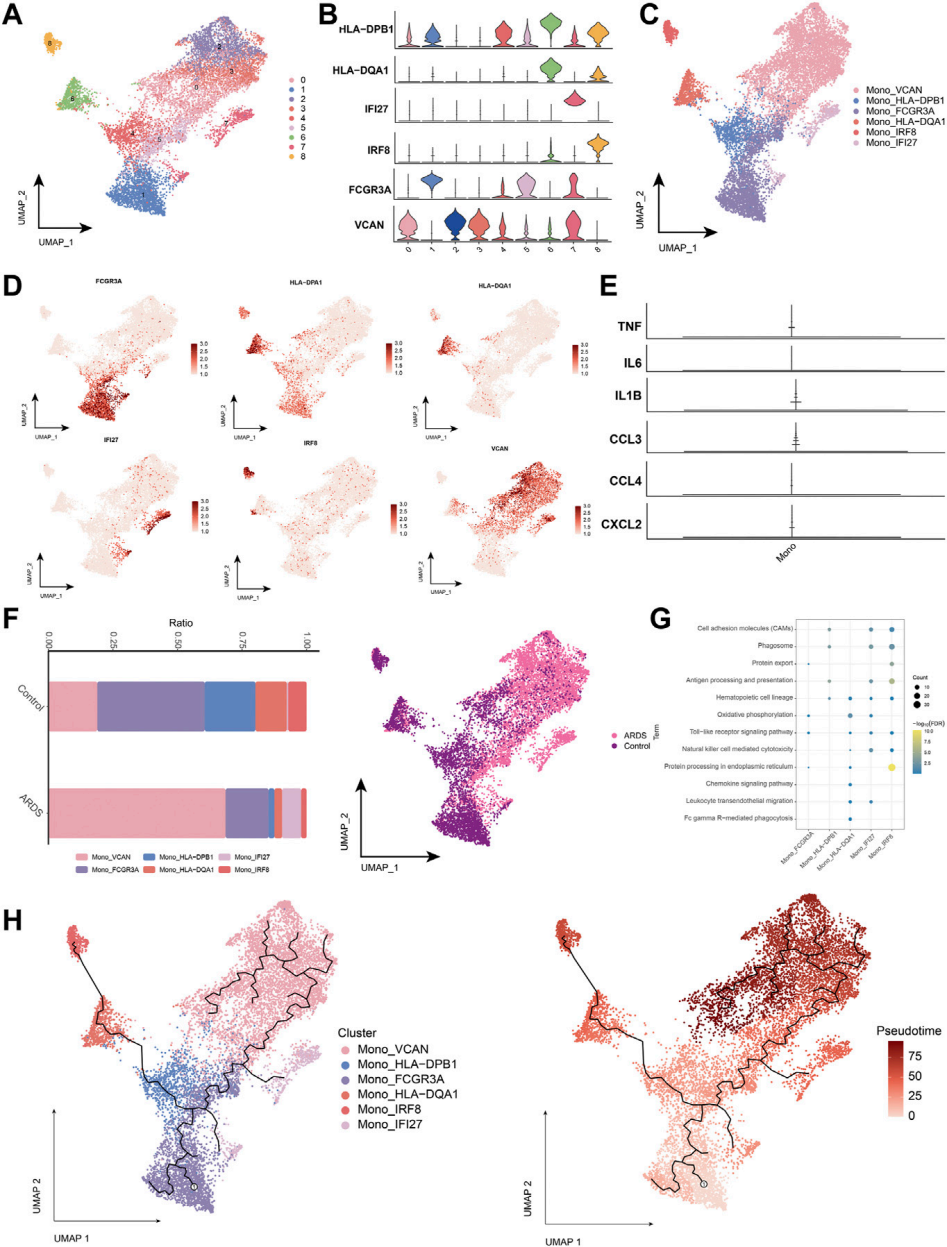
Characterization of monocyte subpopulations in patients with COVID-19-induced ARDS **(A)**. Single-cell atlas demonstrating monocyte subpopulations. **(B)**. Cellular markers expressed by monocyte subpopulations. **(C)**. UMAP profiles are colored by annotated cell markers in monocytes. **(D)**. Single cell atlas mapping marker. **(E)**. Previously reported gene expression of inflammatory cytokines produced by monocytes in the peripheral circulation, namely, TNF, IL6, IL1B, CCL3, CCL4 and CXCL2. **(F)**. Differences in monocyte abundance in PBMC of healthy controls and COVID-19-induced ARDS patients. **(G)**. Signaling pathways significantly involved in monocyte subpopulations. **(H)**. Pseudotime analysis demonstrating proposed chronological values and pseudotime trajectories of monocyte subpopulations.

Next, we analyzed monocytes in more detail, as these cells appeared associated most strongly in patients with COVID-19-induced ARDS (Figures 3A,B). The major monocyte subpopulations, including six subpopulations, were identified on the expression of typical genes (Figures 3C,D). We also examined the expression of previously reported inflammatory cytokines (*TNF, IL6, IL1B, CCL3, CCL4* or *CXCL2*) produced by circulating monocytes in patients with COVID-19. Notably, we did not find peripheral monocytes that abundantly expressed the pro-inflammatory cytokine genes *TNF, IL6, IL1B, CCL3, CCL4* or *CXCL2* (Figure 3E). This suggests that peripheral monocytes may not contribute to the putative cytokine storm in COVID-19-induced ARDS. Subpopulations of Mono_FCGR3A (CD16 monocytes expressing FCGR3A), Mono_HLA-DPB1, and Mono_HLA-DQA1 were depleted in patients with COVID-19-induced ARDS. Furthermore, Mono_VCAN and Mono_IFI27 subpopulations were significantly upregulated in patients with COVID-19-induced ARDS (Figure 3F), indicating that CD16 monocytes were depleted and HLA class II was downregulated in patients with COVID-19-induced ARDS with typical inflammatory features.

We found that cell adhesion molecules, the mitogen-activated protein kinases signaling pathway, and the hematopoietic cell lineage were involved in the chemokine signaling pathway (Figure 3G). To further decipher the transcriptional changes that occurred in monocytes during the transition from a healthy state to a diseased condition, we performed pseudo-temporal inference using Monocle3 with UMAP embedded in a two-dimensional space of monocyte subpopulations (Figure 3H). The pseudo-time tree revealed a continuous trajectory of monocytes from healthy to diseased states, which correlated with the pseudo-time values.

### 3.3 Characterization of CD8^+^ T cell subpopulations in patients with ARDS induced by COVID-19

**FIGURE 4:**
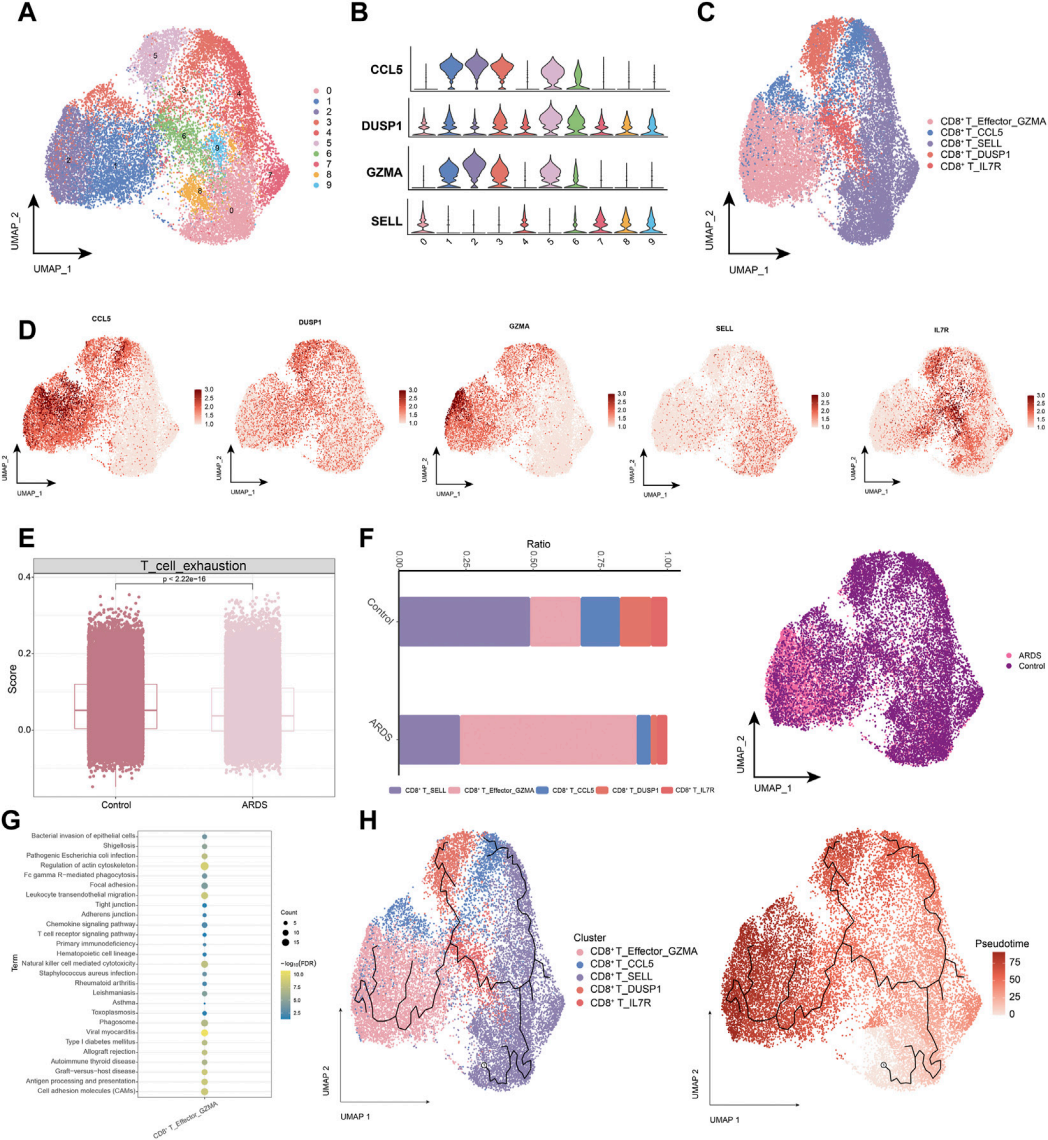
Characterization of CD8^+^ T cell subpopulations in ARDS patients induced by COVID-19 **(A)**. Single-cell atlas demonstrating CD8^+^ T cell subpopulations. **(B)**. Cellular markers expressed by CD8^+^ T cell subpopulations. **(C)**. CD8^+^ T cells in the UMAP atlas are colored by the annotated cell marker. **(D)**. Single cell atlas mapping marker. **(E)**. Expression of genes encoding T cell exhaustion-related genes in healthy control donors and COVID-19-induced ARDS patients. **(F)**. Differences in CD8^+^ T cell abundance in PBMC of healthy controls and COVID-19-induced ARDS patients. **(G)**. Signaling pathways significantly involved in CD8^+^ T cell subpopulations. **(H)**. Pseudotime analysis demonstrating proposed chronological values and pseudotime trajectories of CD8^+^ T cell subpopulations.

Next, we analyzed the CD8^+^ T cell subpopulations (Figures 4A,B), and the UMAP plots of CD8^+^ T cells to detect substantial differences in cell phenotypes (Figures 4C,D). As SARS-CoV-2 infection is associated with cytotoxic lymphocyte failure (Diao et al., 2020), we analyzed the gene expression associated with T cell exhaustion in healthy control donors and patients with COVID-19-induced ARDS (Figure 4E). Patients with COVID-19-induced ARDS had a significantly increased proportion of activated-state T cells, including the CD8^+^ T_Effector_GZMA subpopulation (Figure 4F), which were present at high rates in patients with COVID-19-induced ARDS. Specifically, enrichment analysis revealed that the CD8^+^ T_Effector_GZMA subpopulation was significantly involved in leukocyte transendothelial migration, natural killer cell-mediated cytotoxicity, chemokine signaling, and T-cell receptor signaling (Figure 4G). Pseudotime trajectories revealed a continuous trajectory of CD8^+^ T cell subpopulations from health to disease that correlated with pseudotime values (Figure 4H). These data suggest that impaired T-cell responses, in the context of a predominantly preserved humoral immune response, may lead to poorer outcomes in patients with COVID-19.

### 3.4 Characterization of naïve T cell subpopulations in patients with COVID-19-induced ARDS

**FIGURE 5:**
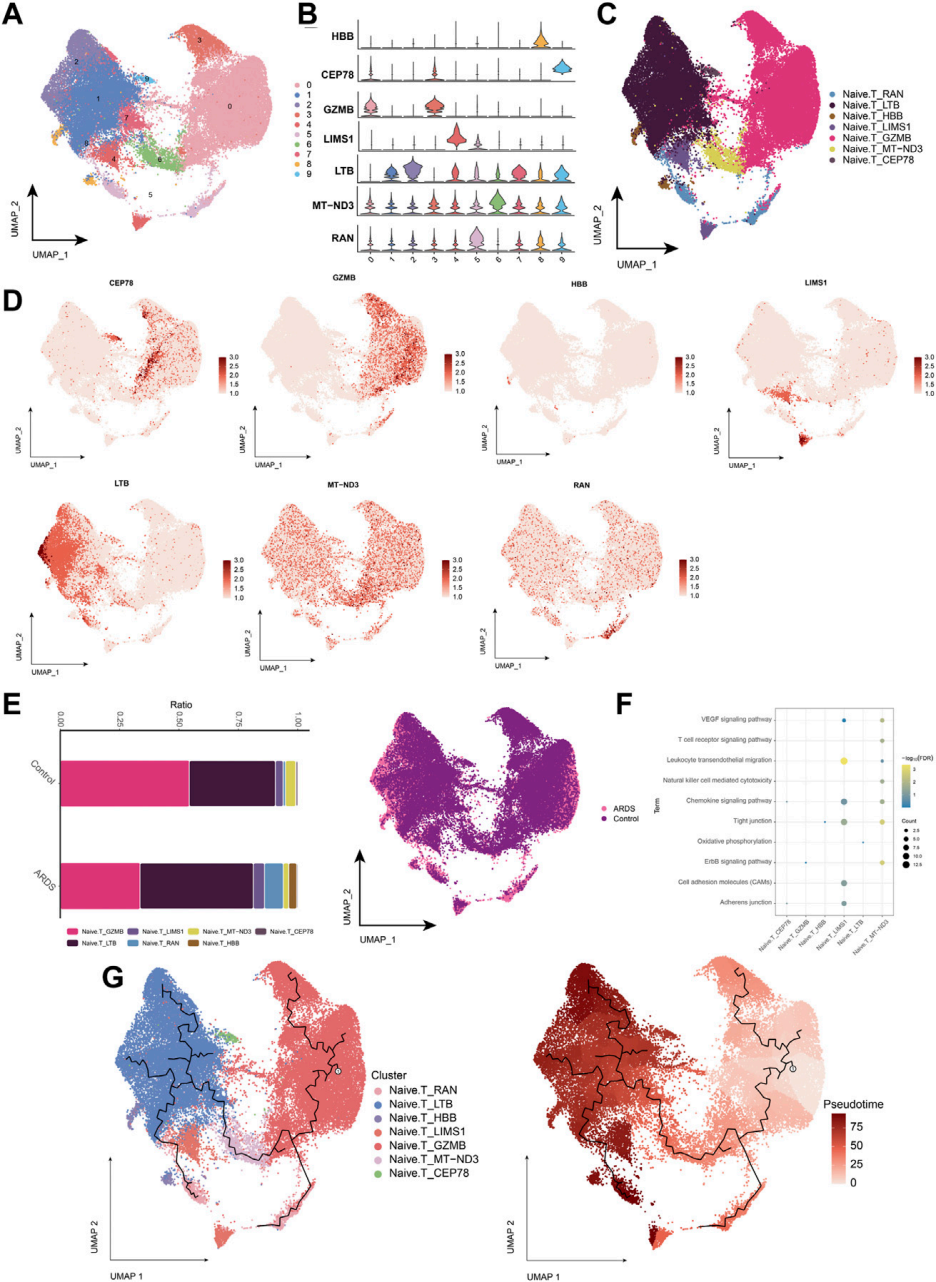
Characterization of COVID-19-induced Naive. T cell subpopulations in ARDS patients **(A)**. Single-cell atlas demonstrating Naive. T cell subpopulations. **(B)**. Cellular markers expressed by Naive. T cell subpopulations. **(C)**. Naive. T cells in the UMAP atlas colored by annotated cell markers. **(D)**. Single-cell atlas mapping the expression of Naive. T cell marker. **(E)**. Differences in Naive. T cell abundance in PBMC from healthy controls and COVID-19-induced ARDS patients. **(F)**. Signaling pathways significantly involved in Naive. T cell subpopulations. **(G)**. Pseudotime analysis showing proposed chronological values and pseudotime trajectories of Naive. T cell subpopulations.

In patients with COVID-19-induced ARDS, immune activation or depletion leads to a reduction in naïve T-cells. Further analysis of naïve T-cell subpopulations (Figures 5A,B) yielded seven different cell subpopulations (Figure 5C), and the major markers were mapped to single-cell profiles (Figure 5D). Similarly, changes in the abundance of naïve T-cell subpopulations were observed in healthy controls and patients with COVID-19-induced ARDS (Figure 5E). Analysis of the enrichment of each subpopulation of naïve T-cells showed that the naïve T-cell subpopulation activated oxidative phosphorylation, antigen processing and presentation, cell adhesion molecules, and chemokine signaling pathways (Figure 5F). Using pseudotime trajectory analysis, we also explored the developmental trajectory of naïve T-cells (Figure 5G).

### 3.5 Characterization of COVID-19-induced B-cell subpopulations in ARDS patients

**FIGURE 6:**
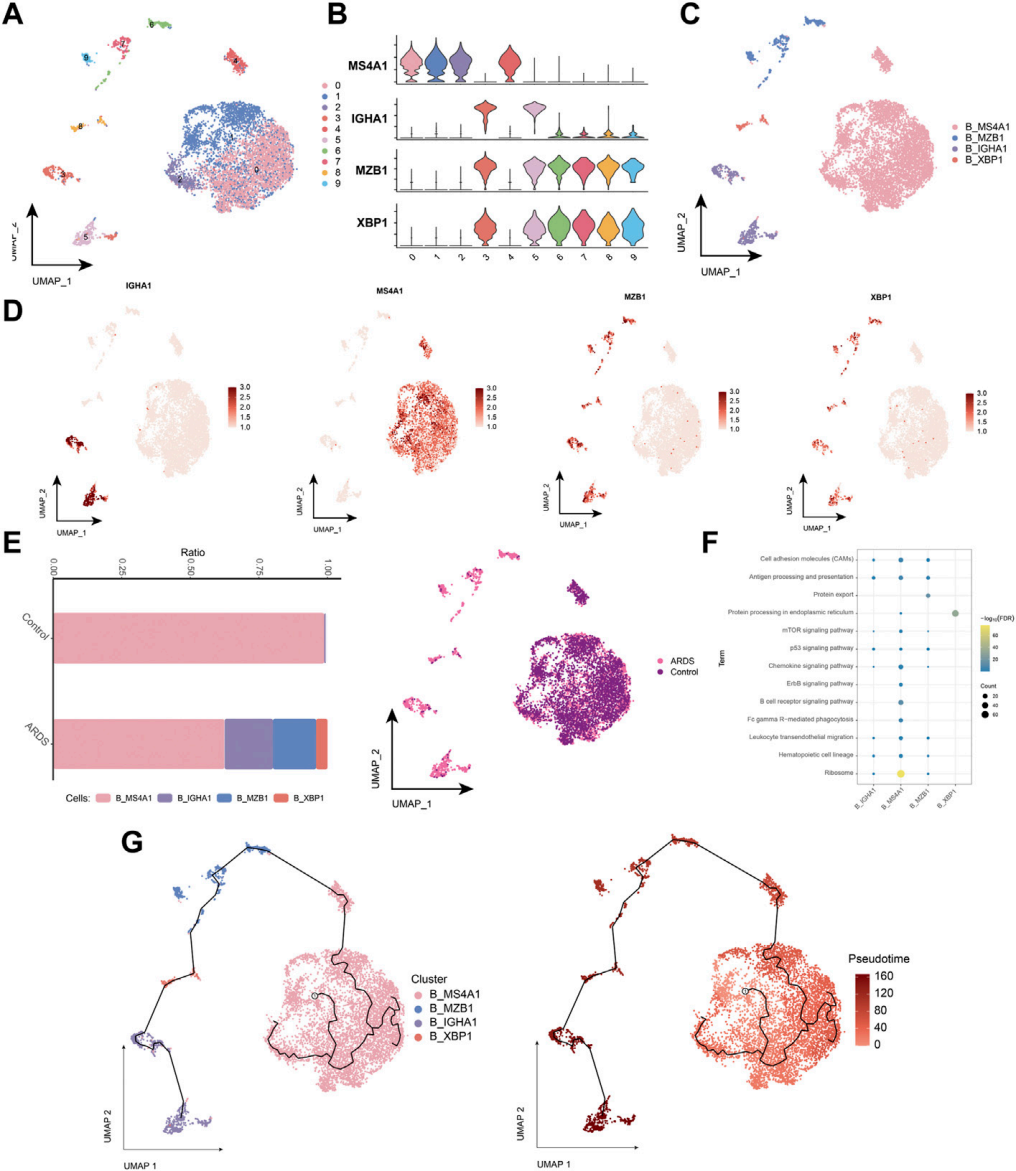
Characterization of COVID-19-induced B-cell subpopulations in ARDS patients **(A)**. Single-cell atlas demonstrating B-cell subpopulations. **(B)**. Cellular markers expressed by B-cell subpopulations. **(C)**. UMAP mapping in B cells colored by annotated cell markers. **(D)**. Single-cell atlas mapping the expression of B-cell markers. **(E)**. Differences in B-cell abundance in PBMC from healthy controls and COVID-19-induced ARDS patients. **(F)**. Signaling pathways significantly involved in B-cell subpopulations. **(G)**. Pseudotime analysis demonstrating proposed chronological values and pseudotime trajectories of B-cell subpopulations.

B cells were subdivided into four subpopulations of B-lymphocytes (Figures 6A,B), and B cells into 4 cell subpopulations (Figures 6C,D). Compared to healthy individuals, the proportion of the memory B-cell subpopulation (B_MS4A1) was significantly lower and the proportion of the plasma cell (Plasma B) subpopulation (B_MZB1) was higher in patients with COVID-19-induced ARDS (Figure 6E). Further exploration of the biological pathways involved in B-cell subpopulations revealed that these B-cell subpopulations were significantly involved in the B-cell receptor signaling pathway, chemokine signaling pathway, hematopoietic cell lineage, and oxidative phosphorylation (Figure 6F). The pseudo-time trajectory revealed a continuous trajectory of B-cell subpopulations from healthy to diseased that correlated with pseudo-temporal values (Figure 6G). These results revealed the transcriptomic profile of the B-cell subpopulation in COVID-19-induced ARDS patients. Here, memory B cells and plasma cells differed significantly between healthy controls and patients with COVID-19-induced ARDS. Memory B cells were reduced and plasma cells were increased in patients with COVID-19-induced ARDS. In the proposed time trajectory (Figure 6G), memory B cells are in the early stage of development, and plasma cells are in the late stage of development, suggesting that memory B cells specifically recognize antigens and subsequently proliferate and differentiate to produce plasma cells.

### 3.6 Strong cytokine communication between T cells, monocytes, and B cells

Further intercellular communication analysis of cell subpopulations revealed strong cytokine communication among T cells, monocytes, and B cells (Supplementary Figure S1). In particular, T cell subpopulations target receptors on other cells via CCL5 and may play an important role in patients with COVID-19-induced ARDS.

## 4 Discussion

The pathogenesis of COVID-19-associated ARDS involves complex interactions between underlying cellular and molecular mechanisms that are not yet fully understood (Fan et al., 2020). Mapping the immune cell profile of COVID-19-induced ARDS is critical for elucidating the pathogenesis of this disease. Our study explores the immunological profile of patients with COVID-19-induced ARDS to understand the genotype-phenotype relationship.

Patients with COVID-19-induced ARDS exhibited a consistent and intense inflammatory response, which was predominantly present in the patients’ PBMC. Furthermore, extensive immune activation was observed in patients with COVID-19-induced ARDS, as evidenced by an increased proportion of monocytes and B cells and a decreased proportion of T cells. CD8^+^ T-cells exhibited higher cytotoxicity in patients with COVID-19-induced ARDS. It has been documented that in severe COVID-19 infection, a cytokine storm can develop alongside excessive monocyte and macrophage activation, leading to ARDS. Moreover, B cells increased in patients with COVID-19-induced ARDS, suggesting that high activation of humoral immunity is a typical feature of the immune response in patients with COVID-19-induced ARDS. CD8^+^ T cells are significantly less abundant in patients with COVID-19-induced ARDS, as previously reported (Huang et al., 2020; Liu et al., 2020; Zhou et al., 2020). T cells control overactive innate immune responses (Kim et al., 2007), and the depletion of CD8^+^ T cells may enhance innate immune responses over long periods, which is consistent with the findings of Zhou et al. (Zhou et al., 2020). Therefore, we suggest that this attenuated adaptive response, as well as an overactivated innate immune inflammatory response, may increase mortality in patients infected with COVID-19. Moreover, we observed an increase in CD14^+^ monocytes and a decrease in CD16^+^ monocytes in patients with COVID-19-induced ARDS. Another study reported that patients with COVID-19-induced ARDS had a higher proportion of CD14^+^ monocytes, accounting for approximately 50% of the total number of cells (Liu et al., 2020). HLA class II genes were increased in monocytes, which is consistent with previous studies (Li et al., 2021). In patients with COVID-19-induced ARDS, we found that T cells activated innate immune responses, defense responses to viruses, responses to type I interferons, and type I interferon-related signaling pathways compared to healthy controls.

Recent studies have shown that chemokines also play a key role in COVID-19 and are associated with cytokine storms in patients (Zhang et al., 2020b). Results showed that patients with COVID-19 who were admitted to intensive care units had higher concentrations of CXCL10, CCL2, and TNF-α compared to that in patients with milder infections (Xu et al., 2020). Cytokine storms can easily affect the immune system, leading to ARDS, multi-organ failure, and even death (Xu et al., 2020). Additionally, a cytokine storm leads to more severe processes. Interferons, interleukins, chemokines, and TNFs are the main factors in the development of cytokine storms in patients with COVID-19. Early T cell responses play a crucial role in viral clearance during acute respiratory infections. Additionally, cytokine storms may influence the severity of coronavirus by reducing the number of T cells (Diao et al., 2020). In this study, CD8^+^ T cells overexpressed their cytokines, causing an excessive inflammatory response, which may be a key cause of the cytokine storm. Many inflammation-associated pathways were enriched in the subpopulations of specifically expressed cells.

Despite these findings, our study had some limitations. Our sample size was small, only the peripheral blood was evaluated, and patients had different times of clinical presentation; these factors may have had affected the transcriptional landscape. As disease severity was not differentiated between samples, we were unable to directly compare the cellular alterations present in patients with different disease severities. As the number of T cells may be a determinant of successful viral clearance, future studies should focus on the relationship between T cell number and disease severity.

## 5 Conclusion

In summary, we found that the immunology of healthy controls and patients with COVID-19-induced ARDS was significantly different. Notably, there was a decrease in the number of T cells and an increase in the number of B cells and monocytes, which resulted in an abnormal peripheral adaptive immune response.

## Data Availability

The datasets presented in this study can be found in online repositories. The names of the repository/repositories and accession number(s) can be found below: National Genomics Data Center under HRA004752.
