# Supplementary figures and images for: Documenting the immune response in patients with COVID-19-induced acute respiratory distress syndrome
Source: Front Cell Dev Biol. 2023 Jun 9;11:1207960. doi: 10.3389/fcell.2023.1207960 (PMC10288867; doi:10.3389/fcell.2023.1207960)

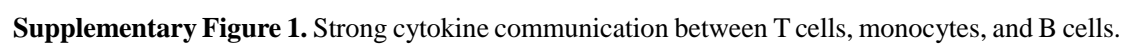

Supplement: Supplementary file 3 [file DataSheet1.PDF]
